# Breast Cancer Screening Program in Lithuania: Trends in Breast Cancer Mortality Before and During the Introduction of the Mammography Screening Program

**DOI:** 10.15388/Amed.2020.27.2.3

**Published:** 2020-12-23

**Authors:** Laura Steponavičienė, Rūta Briedienė, Rasa Vansevičiūtė-Petkevičienė, Daiva Gudavičienė-Petkevičienė, Ieva Vincerževskienė

**Affiliations:** Outpatient Department, National Cancer Institute, Vilnius, Lithuania; Department of Radiology, National Cancer Institute, Vilnius, Lithuania Faculty of Medicine, Vilnius University, Lithuania; Outpatient Department, National Cancer Institute, Vilnius, Lithuania Faculty of Medicine, Vilnius University, Lithuania; Department of Breast Surgery and Oncology, National Cancer Institute, Vilnius, Lithuania; Laboratory of Clinical Oncology, National Cancer Institute, Vilnius, Lithuania

**Keywords:** breast cancer, mortality, mammography screening program, cancer registry

## Abstract

**Abstract. Background.:**

Breast cancer is the most frequent oncological disease as well as the leading cause of cancer death among women worldwide. Decline in mortality in economically strong countries is observed. This decline is mostly related to early diagnosis (an improvement in breast cancer awareness and the mammography screening program (MSP)) and a more effective treatment. In the end of 2005, MSP started in Lithuania. The main aim of this article is to evaluate the breast cancer mortality during 22 years in Lithuania, as well as changes before the start of the MSP and during its implementation, in order to assess the influence of the MSP on mortality.

**Materials and methods.:**

Analysis is based on data from the population-based Lithuanian Cancer Registry. Analysis of changes in mortality includes the period from 1998 to 2019. Age standardized mortality rates are calculated for assessment of changes. Joinpoint regression analysis is used.

**Results.:**

Applying the segmental regression model, it was found that during the study period mortality was statistically significantly decreasing by -1.1% each year. Mortality among women under the age of 50 decreased both before and during the implementation of MSP. Mortality in the target population also was already decreasing until the implementation of the program, but a significant reduction in mortality was observed in this group since 2006.

**Conclusions.:**

Overall breast cancer mortality is decreasing in Lithuania. After the implementation of MSP the largest reduction in mortality was observed in the target population, however, it is not as pronounced as it could be with the well-organized MSP.

## Abstract (lt)

**Krūties vėžio patikros programa Lietuvoje: krūties vėžio mirtingumo tendencijos prieš pradedant ir atliekant mamografijos patikros programą:**

**Santrauka.**

Krūties vėžys yra dažniausia onkologinė liga ir pagrindinė moterų mirties nuo vėžio priežastis visame pasaulyje. Ekonomiškai stipriose šalyse mirtingumas nuo šios ligos mažėja daugiausia dėl pagerėjusios ankstyvos diagnostikos (krūties vėžio žinomumo padidėjimas ir atrankinės mamografinės patikros (AMP) įdiegimas) ir efektyvesnio gydymo. 2005 m. pabaigoje Lietuvoje pradėta vykdyti AMP. Pagrindinis šio straipsnio tikslas – įvertinti mirtingumą nuo krūties vėžio per pastaruosius 22 metus Lietuvoje, taip pat jo pokyčius prieš AMP įdiegimą ir jos vykdymo metu, siekiant įvertinti AMP įtaką mirtingumui.

**Medžiaga ir metodai.**

Analizė pagrįsta populiacinio Vėžio registro duomenimis. Mirtingumo pokyčių analizė apėmė laikotarpį nuo 1998 iki 2019 metų. Pokyčiams įvertinti buvo apskaičiuoti pagal amžių standartizuoti mirtingumo rodikliai. Mirtingumo pokyčių analizė atlikta naudojant segmentinės regresijos metodą.

**Rezultatai.**

Naudojant segmentinės regresijos metodą nustatyta, kad tiriamuoju laikotarpiu mirtingumas statistiškai reikšmingai mažėjo kiekvienais metais po 1,1 procento. Moterų iki 50 metų mirtingumas mažėjo tiek prieš AMP įdiegimą, tiek jos vykdymo metu. Tikslinės populiacijos moterų mirtingumas taip pat mažėjo iki programos įgyvendinimo, tačiau nuo 2006  m. šioje grupėje pastebimas reikšmingas mirtingumo sumažėjimas.

**Išvados.**

Bendras mirtingumas nuo krūties vėžio Lietuvoje mažėja. Įgyvendinus AMP, labiausiai mirtingumas mažėjo tikslinės populiacijos moterų amžiaus grupėje, tačiau jis nėra toks ryškus, kaip galėtų būti vykdant tinkamai organizuotą AMP. Norint išaiškinti tikrą Lietuvoje vykdomos AMP poveikį mirtingumui, reikia tolesnių išsamesnių tyrimų.

**Raktažodžiai:** krūties vėžys, mirtingumas, mamografinės patikros programa, vėžio registras

## Introduction

Breast cancer (BC) is the most frequent oncological disease, also it is the leading cause of cancer death among women in 101 countries worldwide [1]. It is estimated that in 2018 there were 626,679 deaths worldwide due to breast cancer in women. This represents 15% of all cancer deaths among women [2]. During the last three decades a decline in mortality in economically strong countries is observed. According to the opinion of many authors, this decline is most related to early diagnosis (an improvement in breast cancer awareness and the mammography screening program (MSP)) and a more effective treatment [1,3,4], although each investigator indicates different impact of each of these factors. The BC mortality is projected to decline by 7–23% for the ongoing MSP, and by 12–21% for a more effective treatment [5].

In Europe, BC also is the most frequently diagnosed neoplasm in women and the leading cancer site for deaths from cancer in women [6]. The decline in mortality from BC is seen in many European countries, especially in the Western part of Europe [7]. The improvement in survival in Europe since 1990 is thought to be mainly due to the ongoing changes in the diagnosis and treatment of BC: the implementation of MSP in many countries, the introduction of effective hormonal therapy and chemotherapy, and the progress of radiation and surgery [8,9]. However, it is emphasized that the implementation of MSPs, the availability of innovative treatments and the cost of health care vary considerably across European countries, which may contribute to uneven mortality reduction [7].

The primary goal of MSP is to reduce the BC mortality. MSP is a complex multistep process therefore it is very important to monitor the performance of the national BC screening program from its inception to determine how closely the benefits it achieves approach the benefits seen in the randomized trials and population demonstration projects [10].

In the end of 2005, the MSP started in Lithuania. In a recent report on cancer screening in the EU, Lithuania was the country with the lowest participation rate (44.9% in 2014) and one of three countries, where centralized invitation through a screening registry was not implemented [11]. Despite the fact that MSP has been running for almost 15 years there has been no work assessing the impact of MSP on mortality. 

The main aim of this article is to evaluate the breast cancer mortality during 22 years in Lithuania, as well as changes before the start of MSP and during its implementation, in order to assess the influence of MSP on mortality. 

## Material and methods

### Data sources

Analysis is based on data from the population-based Cancer Registry. The Lithuanian Cancer Registry is a population-based cancer registry that contains personal and demographic information (place of residence, sex, date of birth and vital status), as well as information on the diagnosis (cancer site, date of diagnosis and method of cancer verification) and death (date of death and cause of death) of all cancer patients in Lithuania, where the population size is approximately 3 million residents according to the 2011 census [12]. The principal sources of information on cancer cases are primary, secondary and tertiary health care institutions in the country that are responsible for providing notification when cancer is diagnosed. All physicians, all hospitals and other institutions in the country must send a notification to the Lithuanian Cancer Registry of all cancer cases that come to their attention. The notifications, which are supplemented by the death certificate information, are built into a database suitable for statistical use. Lithuanian Cancer Registry contains information on all cancer cases diagnosed in Lithuanian residents since 1978. Since the period 1988–1992, the Registry data have been included in the ‘Cancer Incidence in Five Continents’ [13].

### Breast cancer screening program

The Lithuanian BC screening program started at the end of 2005, when the order of the Lithuanian Health Ministry was issued. According to the program, the target population is defined as 50-69-year-old women. Women are referred to screening mammography by their general practitioners or gynaecologists every two years. Mammograms are obtained in two standard projections (craniocaudal and mediolateral oblique) and are independently read by two radiologists. Both screen-film and digital mammography systems are present in Lithuania. For reporting of screening and additional imaging results, the BIRADS (*Breast Imaging Reporting and Data System*) system is used, and for the evaluation of breast density, typology according to the ACR (*American College of Radiology*) is included [14]. The assessment information is sent to the general practitioners within two weeks and they inform women about the results of mammography. 

### Statistical analysis

Analysis of changes in mortality included the period from 1998 to 2019. The aim was to assess trends in the overall breast cancer mortality and changes before the start of MSP implementation and during its course. Women were divided into 3 age groups: from 0 to 49 y.o.; from 50 to 69 y.o. (the target population summoned to take part in MSP); 70 y.o. and older, aiming to assess the influence of MSP on mortality in the target population. The period of research was divided into 2 periods: a period before MSP (1998 to 2005) and a period of MSP implementation (from 2006 to 2019). Age standardized mortality rates were calculated for assessment of changes (the direct standardization method was applied and the old European Standard Population was used). Analysis of the breast cancer mortality was carried out by using JOINPOINT software (version 4.3.1.0). Joinpoint regression analysis was used to identify points where a statistically significant change over time in the linear slope of the trend occurred. The annual percent change (APC) was calculated for the trends by means of the generalized linear model. APCs were considered statistically significant if p < 0.05. Joinpoint analysis was performed for all ages combined and age-specific rates. The graphic depiction was implemented by using Microsoft Excel software.

## Results

**Fig 1.**
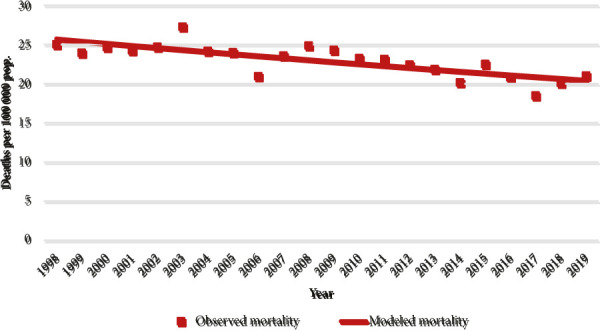
Breast cancer mortality trend in Lithuania, 1998–2019. All ages.

**Fig 2.**
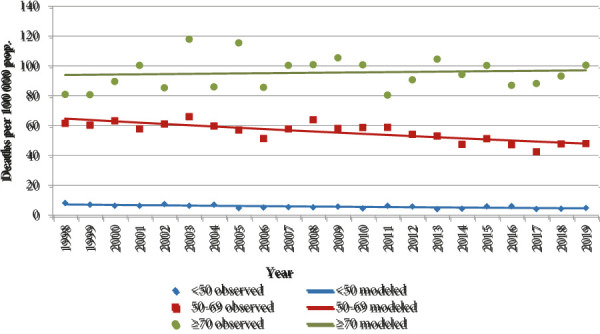
Breast cancer mortality trends by age group in Lithuania, 1998–2019

The age standardized mortality rate in 1998 was 25.1/100 000, and in 2019 it was 21.0/100 000. During the study period, the overall breast cancer mortality was statistically decreasing by -1.1% each year (95% CI -1.2;-0.9). The significant decrease in mortality was observed in both younger age groups, but in the group older than 70 y.o. the nonsignificant increase by 0.2% was found (95% CI -0.1; 0.4). Mortality rates and their changes are presented in Table 1 and Fig. 1. and Fig. 2.

**Table 1.** Number of breast cancer deaths, age standardized mortality rates and annual percent changes in mortality trends by age group in Lithuania, 1998–2019

Age group	1998		2019		Annual percent change	95% confidence intervals	
	Number of deaths	Age standardized mortality rate	Number of deaths	Age standardized mortality rate		Upper	Lower
**All ages **	534	25.1	550	21.0	-1.1	-1.2	-0.9
**< 50 **	103	5.8	47	4.9	-2.1	-2.4	-1.8
**50-69 **	266	61.6	211	48.0	-1.4	-1.6	-1.3
**≥ 70 **	165	80.9	292	100.5	0.2	-0.1	0.4

Before the implementation of MSP the mortality decrease was seen in women younger than 50 y.o., while the decrease in the target population was not statistically significant, and in the age group older than 70 y.o. mortality was distinctly increasing. After the implementation of MSP the mortality rates have decreased in all age groups. The largest reduction in mortality was observed in the target population. In the age group older than 70 y.o. the decrease was not statistically significant. Changes in mortality in age groups in two periods (before the start of MSP and after its implementation) are presented in Table 2.

**Table 2.** Annual percent changes in breast cancer mortality trends by age group before (1998–2005) and after (2006–2019) the start of MSP in Lithuania

Age group	1998-2005			2006-2019		
	Annual percent change	95% confidence intervals		Annual percent change	95% confidence intervals	
		Upper	Lower		Upper	Lower
**All ages **	0.1	-0.4	0.7	-1.3	-1.6	-1.0
**< 50 **	-3.8	-5.2	-2.4	-1.0	-1.7	-0.3
**50-69 **	-0.4	-1.0	0.1	-2.1	-2.4	-1.7
**≥ 70 **	4.5	3.1	6.0	-0.2	-0.6	0.2

## Discussion

Several countries started implementing national MSPs in 1980s and 1990s, based on the results of randomized trials and meta-analyses, that have shown that the breast cancer mortality can be reduced by 25-30% with mammography screening [15,16]. However, there is still a debate about the extent to which MSP can affect the BC mortality. Some researchers say that it is not clear how much this effect would persist outside the controlled trial environment. Cochrane review published in 2001 challenged the results of some randomized controlled trials leading to a major controversy on the benefits of mammography screening [17-19]. On the other hand, the extensive review conducted in Canada concluded that modern BC screening programs may achieve greater reduction in the BC mortality than it was found in the screening trials [20]. This only further underlines the importance for each country to assess its own performance indicators.

In order to assess the effectiveness of MSP implementation in the country and its impact on mortality, it is necessary to assess trends in the BC mortality in the country. Such studies evaluate the changes in mortality in the population after the introduction of MSP. For comparison, the BC mortality before and after the introduction of screening is assessed, as well as and the trends in age groups affected and unaffected by screening [21].

Applying the segmental regression model, it was found that mortality among women under the age of 50 decreased both before and during the implementation of MSP. Mortality in the target population also was already decreasing until the implementation of the program, but since 2006 a significant reduction in mortality was observed in this group. Thus, in our country, mortality decreased both in the target population exposed to MSP and in the group of women under 50 who are not included into the program. In the group of women over the age of 70, the overall increase in mortality since 1998 was seen. Since 2006, the reduction in mortality was observed in this age group, but it was not statistically significant. Women in this age group could be indirectly exposed to MSP (early detection of a tumour before the age of 70 years and successful treatment should reduce mortality in this age group as well) [22].

One study examined trends in the BC mortality in thirty European countries from 1989 to 2006 [7]. Mortality from BC decreased by an average of 19% during the study period. However, these figures ranged from 45% reduction in Iceland up to 17% increase in Romania. Mortality from BC reduced by ≥ 20% in fifteen countries and this decline was highest in those countries with the highest mortality rates. England and Wales, Northern Ireland, and Scotland had the highest reductions in mortality, at 35%, 29% and 30%, respectively. In France, Finland and Sweden, mortality fell by 11%, 12% and 16%, respectively. In Central European countries, mortality did not decrease or even increased during the study period. Mortality declining trends between 1988 and 1996 and a steady decline in mortality from 1999 to 2006 suggests that these trends may continue. Mortality reduction varied amongst age groups: in women <50 years of age – 37% (in different countries from -76% to -14%); in women aged 50-69 – 21% (40% to 14%) and 2% in women ≥ 70 years of age (from - 42% to 80%). The highest decrease in mortality was observed in women younger than 50 years of age. Decrease in mortality among women over 70 years of age was insignificant in this study, and an upward trend in mortality was observed in this age group in 17 Central European countries. In our study, as in the above study, the largest reduction in mortality was observed in the young women age group.

Detailed comparative studies of mortality trends have been performed in Spain, Italy, the Neth-erlands, Denmark and Sweden [23-28]. In some of these studies, a significant reduction in mortality was observed after initiation of MSP, while no such decrease was observed in others, or the decrease occurred independently of the introduction of MSP. Some authors found a decrease in mortality only in the target population, but according to other authors, a decrease in mortality was also observed in age groups not participating in MSP. A review of mortality change studies by Moss and colleagues concluded the reduction in mortality from 1 up to 9% annually for a period of 10–12 years [21]. In our study the reduction in mortality in the target population was quite low, mortality was decreasing only by 2.1% annually and we could expect more substantial decrease with the national-wide MSP which was implemented almost 15 years ago. 

It is quite difficult to determine whether the implementation of MSP had an impact on the reduction of mortality in Lithuania. The fact that a decrease in mortality is also observed in young women not participating in the screening group suggests that there are other factors that contribute to the reduction in mortality. In this group of women, the reduction in mortality is mainly due to improved treatment options [29]. The decrease in mortality observed in Lithuania can be related to the improvement of diagnostics (new radiological equipment was purchased in Lithuania during the study period, radiologist training courses are ongoing) and new treatment options (new hormone therapy, chemotherapy and biological therapy products appeared in Lithuania during the study period and significantly improved treatment outcomes of BC patients).

The decrease in mortality observed in our study among women over the age of 70 during the MSP implementation is not statistically significant and can hardly be attributed to it. These women are indirectly exposed to MSP, a reduction in mortality after some time should also be observed among them [22]. 

There may be several reasons why the impact of the implemented MSP in Lithuania may not be as great as expected. It can be due to a small number of participants and a low number of early stage tumours detected during MSP, as well as a lack of unified system to ensure the most rapid and effective treatment of women diagnosed with BC during MSP. The participation rate in Lithuania seeks only a half of the target population. The cases of BC detected during MSP make only 25% of BC diagnosed in 50-69 year old women. The cases of stage I BC detected during MSP make only 49% of all tumours detected by MSP [30]. Also, the analysis of incidence and stage changes showed that the implementation of the MSP in Lithuania hadn’t influenced on the localized and advanced BC [31].

### Strengths and limitations of the study

While assessing the impact of MSP on mortality, mortality trends over time are usually assessed first. However, it is important to emphasize that it takes a long time for the overall statistics to reflect the impact of MSP on mortality reduction in the general population [21]. It should be mentioned that our study covers a fairly long period (14 years) after the introduction of MSP, in order to evaluate the impact of MSP on mortality.

It should also be noted that the results of trend assessments can be influenced by several factors. Women diagnosed with BC live a long time, with a five-year survival rate of 90% in developed countries [29]. Therefore, the population mortality data also include deaths from BC in women diagnosed with breast cancer before the introduction of MSP that may distort the impact of MSP on mortality rates. In order to elucidate the true impact of MSP in Lithuania on the BC mortality, it would be necessary to conduct a study including only the cases of BC diagnosed after the introduction of MSP.

As in most similar studies comparing the periods before and after the introduction of MSP, in our study the beginning of the second period coincided with the date of introduction of MSP in our country. Such a division of the study into periods may reduce the expected effect of MSP because its effect on population indicators takes time even in case of an effective implementation of MSP. 

As there are many positive developments in the treatment of BC (the increasing use of modern chemotherapy and hormonal drugs), it becomes more difficult to interpret studies of mortality trends.

It is also always difficult to accurately quantify the contribution of other factors to the reduction in mortality. Usually, countries undergo opportunistic screening for BC or pilot projects in individual regions prior to the introduction of an organized MSP at the state level, which also reduces the impact of an organized MSP on mortality changes [30]. As our country does not have a centralized invitation system to participate in MSP and does not have a unified MSP registry, it is not known exactly how many women are checked outside the program.

## Conclusions

During the study period, the overall breast cancer mortality was decreasing statistically significantly. The largest decrease in mortality was observed among women younger than 50 y.o. After the implementation of MSP the largest reduction in mortality was observed in the target population, however, it is not as pronounced as it could be with the well-organized MSP. A further, more detailed research is needed in order to clarify the effects of MSP in Lithuania. 
